# Supplementary material for: Magnetic proximity effect in biphenylene monolayer from first-principles
Source: J Mater Chem C Mater. 2025 Feb 17;13(12):5993–9. doi: 10.1039/d4tc04702h (PMC11843877; doi:10.1039/d4tc04702h)
Supplement: TC-013-D4TC04702H-s001 [file TC-013-D4TC04702H-s001.pdf]

Supplementary Information (SI) for Journal of Materials Chemistry C.
This journal is © The Royal Society of Chemistry 2025

Supporting Information for Magnetic proximity effect in biphenylene monolayer from first-principles

Diego López-Alcalá[a] and José J. Baldoví.[a,*]

[a]Instituto de Ciencia Molecular, Universitat de València, Catedrático José Beltrán 2, 46980 Paterna, Spain.

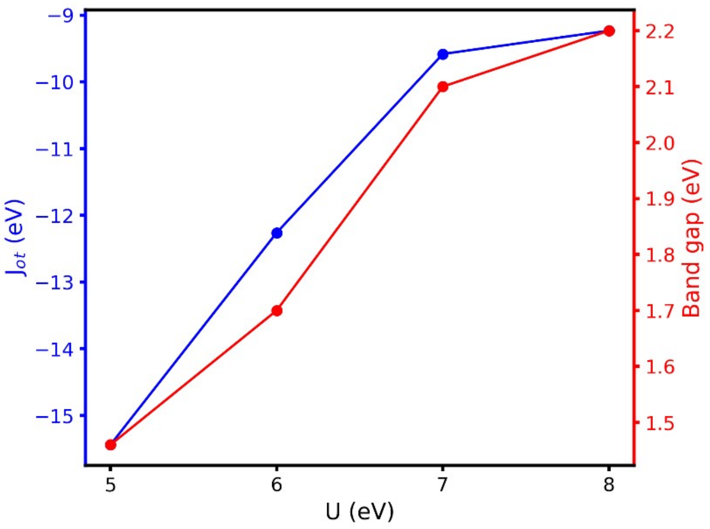

Figure S1: Effect of Hubbard U in dominant magnetic exchange coupling J_{ot} (blue) and in electronic band gap of bulk-like YIG atoms (red).

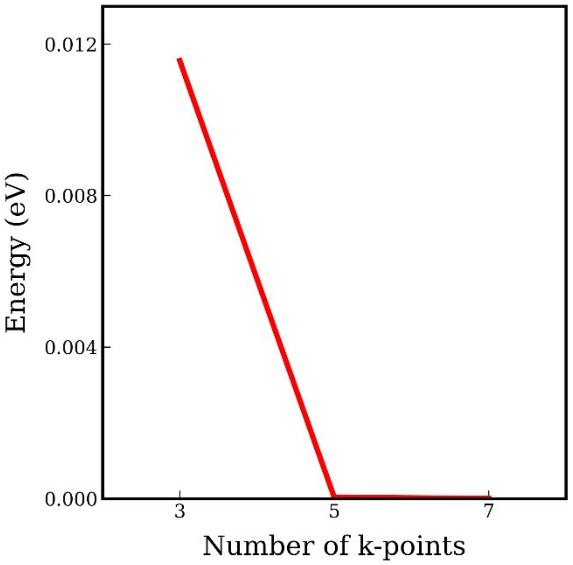

Figure S2: K-point mesh energy convergence.

Table S1: Calculated energy of the possible magnetic configurations of a free standing BPN monolayer.

Magnetic order	Energy (meV)
AFM	0
FM	2.091
Closed - shell	2.090

We computed the charge transfer in the BPN/YIG system using various methods, as summarized in Table S2. The direction of charge transfer is consistent across the results obtained from Bader, Hirshfeld, and Voronoi analyses. Among these, we use the quantitative results from the Bader method for our conclusions, as it provides a more detailed description of the interface, accounting for its intricate topology.[1]

Table S2: Calculated charge transfer in BPN/YIG with different methods.

	Bader	Hidsfrield	Voronoi
**Charge transfer (*e*/f.u.)**	0.58	1.15	1.63

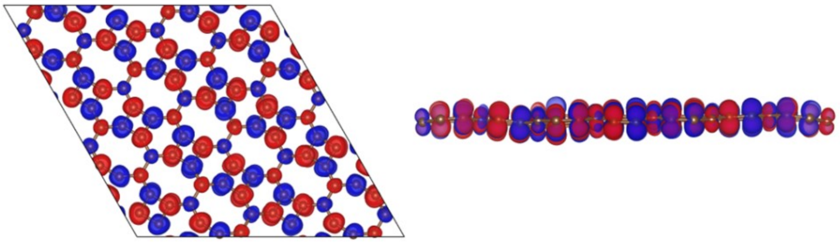

Figure S3: BPN spin density. Isosurface 0.0001 e/Å^3.

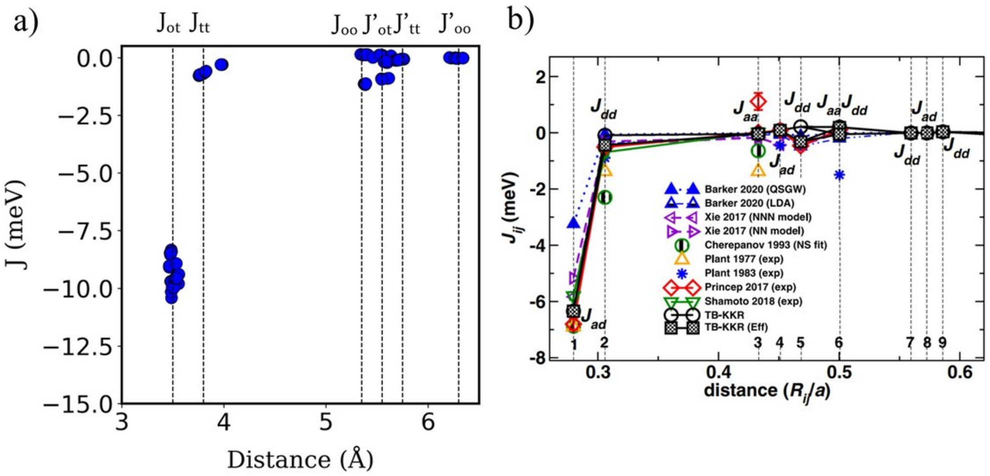

Figure S4: a) Calculated Magnetic exchange couplings (J) in the bulk-like Fe atoms in YIG. **b)** Summary of J in YIG obtained in different works. Obtained from O. I. Gorbatov *et al., Phys. Rev. B,* **104**, 174401 (2021).

Table S3: First neighbor magnetic exchange interaction (J_{ot}) in meV for bulk-like Fe^{O} atoms.

Without BPN	With BPN	Variation (%)
-9.02	-9.46	4.88
-9.08	-9.47	4.23
-9.02	-9.39	4.14
-8.42	-8.62	2.38
-8.34	-8.54	2.39
-8.41	-8.65	2.95
-10.15	-10.29	1.30
-10.41	-10.51	0.95
-9.88	-9.94	0.57
-9.57	-9.72	1.52
-9.43	-9.59	1.67
-9.80	-9.93	1.35
-8.46	-8.59	1.50
-8.54	-8.67	1.58
-9.70	-9.85	1.52
-9.69	-9.90	2.11
-8.48	-8.61	1.59
-9.71	-9.87	1.70

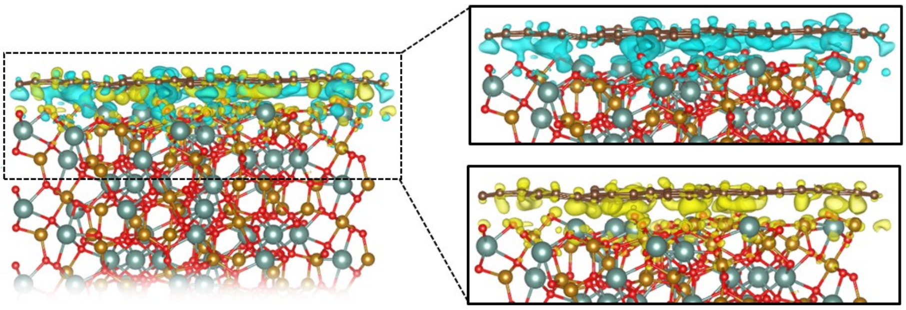

Figure S5: Charge density difference after BPN deposition on (111) YIG surface at $d - d_0 = 0.9$ Å. Color code: blue (yellow) regions represent charge depletion (accumulation). Inset shows separately the charge depletion and accumulation, respectively. Isosurface is set to 0.001 e/Å^3.
